# Immune Signature of Enhanced Functional Avidity CD8^+^ T Cells *in vivo* Induced by Vaccinia Vectored Vaccine

**DOI:** 10.1038/srep41558

**Published:** 2017-02-03

**Authors:** Zhidong Hu, Lingyan Zhu, Jing Wang, Yanmin Wan, Songhua Yuan, Jian Chen, Xiangqing Ding, Chenli Qiu, Xiaoyan Zhang, Chao Qiu, Jianqing Xu

**Affiliations:** 1 Shanghai Public Health Clinical Center, Fudan University, Shanghai, China; 2 Institutes of Biomedical Sciences, Fudan University, Shanghai, China; 3 Key Laboratory of Medical Molecular Virology of MOE/ MOH, Fudan University, Shanghai, China

## Abstract

Functional avidity of T cells is a critical determinant for clearing viral infection and eliminating tumor. Understanding how functional avidity is maintained in T cells is imperative for immunotherapy. However, studies systematically characterize T cell with high functional avidity induced *in vivo* are still lacking. Previously, we and others found vaccinia vectored vaccine (VACV) induced antigen-specific CD8^+^ T cells with relatively high functional avidity to those from DNA vaccine. Herein, we used functional, immune phenotyping and transcriptomic studies to define the immune signature of these CD8^+^ T cells with high functional avidity. Antigen-specific CD8^+^ T cells induced by VACV executed superior *in vivo* killing activity and displayed a distinct transcriptional profile, whereas no significantly differences were found in composition of memory sub-populations and cytokine poly-functionality. Transcriptional analyses revealed unique features of VACV induced CD8^+^ T cells in several biological processes, including transport, cell cycle, cell communication and metabolic processes. In summary, we characterize CD8^+^ T cells of high functional avidity induced *in vivo* by VACV, which not only improves our understanding of adaptive T cell immunity in VACV vaccination, but also provides clues to modulate functional avidity of CD8^+^ T cells for T cell based immunotherapy.

The functional avidity, also termed as antigen sensitivity^1^, is one of the most critical properties that determine T cell functions^2^,^3^. In principle, the strength of stimulus received by T cells upon exposure to defined densities of antigen is determined by their functional avidity. High avidity T cells could recognize virally infected cells at lower surface densities and at an earlier period of infection. Moreover, at given antigen density, T cells with higher levels of avidity could elicit stronger functions^1^,^4^. Thus, high avidity T cells might perform a rapid and readily effector functions at low cognate antigen concentration thresholds, helping effectively eliminate the virus infected cells before mass propagation and viral mutation escapes from immunosurveillance^5^,^6^. Moreover, with wider variant cross-recognition capacity, broader T cell responses and stronger functionality profiles, high functional avidity CD8^+^ T cells also triggered effector functions more readily and undergo promptly expansion *in vivo*, help shaping their immunodominance^4^,^7^,^8^,^9^,^10^,^11^, leading to an efficient viral infected cells clearance^12^,^13^,^14^ and tumor cells elimination^15^,^16^. As a result, the avidity of T cells has been regarded as a major determinant of T cell functionality, control of viral infection and tumor elimination^4^,^7^,^17^,^18^.

It’s well shown that an individual T cell clone can be generated into both high- and low-avidity effectors by *in vitro* stimulation with a high or low concentration of peptide, respectively, which is determined by the initiation of T cell receptor (TCR) signaling^19^,^20^,^21^. Moreover, the Toll-like receptor 8 engagement increased anti-tumor cytotoxic T lymphocyte (CTL) functional avidity *in vitro*^22^. Currently, it’s well demonstrated that T cells could undergo a process called functional avidity maturation when encountered with the antigen, leading to the increased avidity in antigen experienced cells compared to naïve ones^23^. T cell activation signals are believed as the major determinant of functional avidity maturation. However, studies for characterizing factors associated with T cell functional avidity are limited, partially due to the absence of reliable systems eliciting sufficient number of antigen-specific T cells with distinguishable levels of functional avidity.

Consistent with other reports using poxvirus based vaccines^24^,^25^,^26^, we proved that the functional avidity of CD8^+^ T cells primed by DNA vaccination could be enhanced by VACV boost *in vivo*^27^. Importantly, we showed that the intrinsic MyD88 mediated signaling pathway in CD8^+^ T cells, instead of the improvement of antigen presentation efficacy or MyD88 mediated inflammatory milieu, was responsible for this enhancement^27^. However, it’s still not well illustrated that whether the increase of avidity induced by VACV is restricted to a particular model or more broadly applicable. Moreover, immune signature of T cells with differential functional avidity induced *in vivo* by vaccination also remains unknown.

In order to address these questions, we firstly generalized that the ability of boosting functional avidity by VACV with additional immunogens and in mice with distinct genetic background. We then establish a system adoptively transferred with OVA-specific monoclonal TCR transgenic OT-I CD8^+^ T cells, which would provide adequate number of high functional avidity CD8^+^ T cells without interference from TCR diversity. As expected, high functional avidity CD8^+^ T cells derived from this system executed enhanced *in vivo* killing activity and displayed a distinct transcriptional profile, but not correlated with memory phenotype and poly-functionality of antigen-specific CD8^+^ T cells, nor the cytokine profiles of CD4^+^ helper T cells. Finally, global gene expression pattern of VACV induced antigen-specific CD8^+^ T cells showed a unique set of genes which mainly involved in several signaling pathways, compared with DNA vaccination. These results provided a model for the induction CD8^+^ T cells with distinguishable functional avidity *in vivo*, and specialized the characteristics of antigen-specific CD8^+^ T cells with enhanced functional avidity induced by VACV, thus enlighten the understanding of the formulation mechanism of the functional avidity *in vivo*.

## Results

### Enhanced functional avidity of antigen-specific CD8^+^ T cells induced by VACV

**Figure 1:**
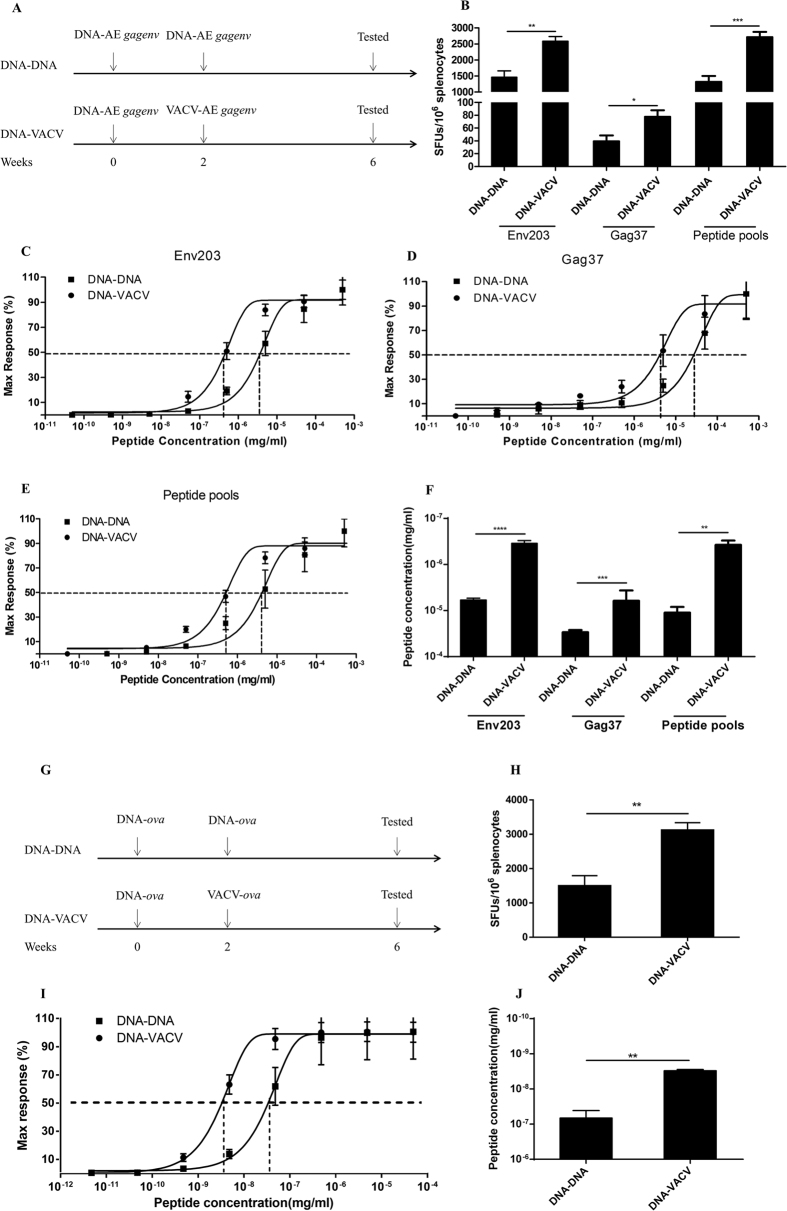
VACV boosted the functional avidity of CD8^+^ T cells primed by DNA vaccination. (**A** to **F**) DNA-VACV regimen induced higher levels of frequency (**B**) and functional avidity of antigen-specific T cell responses against immune dominant (**C**), subdominant epitopes (**D**) and peptide pools (**E**) in a C57BL/6 model using HIV-1 AE Gag-Env as antigen. The summarized EC_50_ of peptide concentration required for IFN-γ production are shown in (**F**). (**G** to **J**) VAVC induced higher levels of frequency (**H**) and functional avidity (**I**) in an OVA-based vaccine mice model. The EC_50_ was shown in (**J**).

Previously, we showed that VACV significantly enhanced the functional avidity of antigen-specific CD8^+^ T cells primed by DNA using HIV-1 CN *gag* as immunogen in a BALB/c mice model^27^. In this study, we generalized this observation with epitopes from additional antigens and in mice with a distinct genetic background. Vaccines expressing HIV-1 AE Gag-Env fusion protein were used to inoculate the C57BL/6 mice at 2 weeks apart (Fig. 1A). As shown in Fig. 1B, irrespective of the epitopes examined in ELISpot assay, DNA prime-VACV boost (DNA-VACV) consistently induced higher levels of antigen specific T cells when compared with DNA prime-DNA boost (DNA-DNA) vaccination. In particular, these VACV boosted cells had enhanced functional avidity, as determined by either immune dominant epitope Env203 (Fig. 1C,F), immune sub-dominant epitope Gag37 (Fig. 1D,F), or AE Gag-Env peptide pools that evaluate T cells recognize all epitopes presented in Gag-Env protein (Fig. 1E,F). This attention was further confirmed by using vaccines expressing OVA (Fig. 1G–J), which is a classical experiment system for studying vaccine induced immune responses. Collectively, these data warranted that VACV could enhance the functional avidity of antigen-specific T cells primed by DNA vaccination, which is not restricted to a particular model but more broadly applicable.

**Figure 2:**
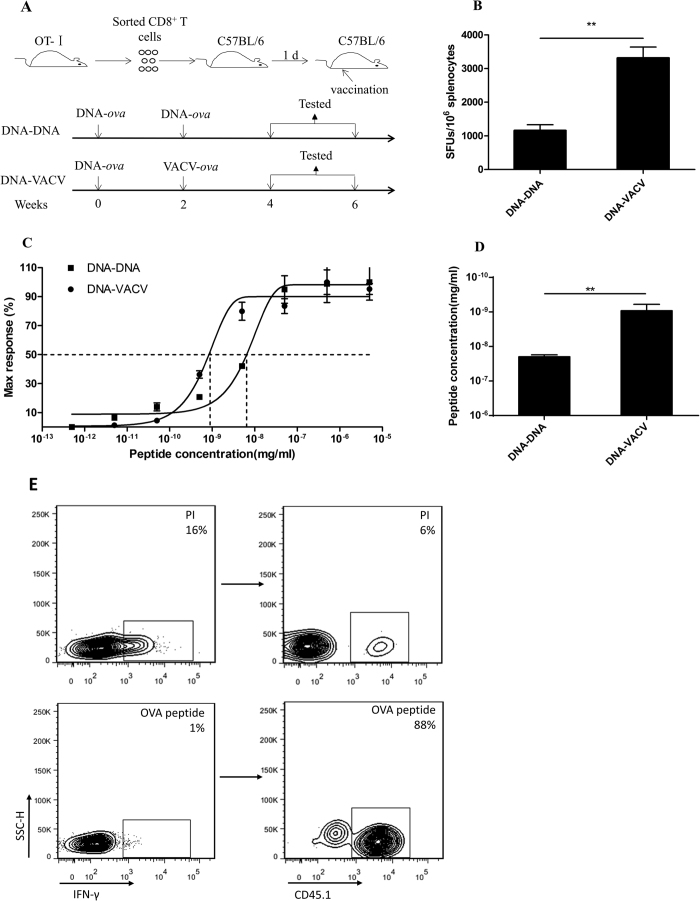
Increased antigen-specific T cell responses with enhanced functional avidity following VACV in an adoptively transferred mice model using OVA as antigen. To exclude the effect of TCR selection, the TCR transgenic OT-1 mice was introduced, the purified monoclonal TCR OT-I CD8^+^ T cells were adoptively transferred into wild type C57BL/6 mice and immunized with OVA antigen as scheduled in (**A**), (**B** to **D**) VACV boost could induce stronger antigen-specific T cell responses (**B**) with higher functional avidity (**C**). The EC_50_ of peptide concentration required for IFN-γ production was shown in (**D**). (**E**) To verify the feasibility of this adoptive transfer model, we calculated the percentage of CD45.1^+^ in IFN-γ^+^ cells after OVA peptide stimulation, data showed that the proportion was almost 90% whereas only 6% by PMA/ionomycin non-specific stimulation, demonstrating that the OVA-specific immune response was dominantly triggered by the exogenous TCR transgenic CD8^+^ CD45.1^+^ T cells instead of the endogenous polyclonal population.

T cells recognize the same epitope bears various TCR. The affinity between TCR and MHC-peptide is one of the critical determinants of functional avidity. In order to control the interference of TCR diversity, we established a model by adoptively transferring the monoclonal CD8^+^ T cells from TCR transgenic OT-I mice into wild type C57BL/6 mice (Fig. 2A). After vaccination, we compared the functional avidity between DNA-DNA and DNA-VACV vaccination. As expected, the differences in both the magnitude (Fig. 2B) and the functional avidity (Fig. 2C,D) of OVA-specific T cells were significant between these two vaccination regimens. As expected, flow cytometric analysis confirmed that the transferred OT-I T cells (CD45.1^+^) was overwhelming to endogenous T cells (CD45.1^−^) recognizing OVA (Fig. 2E). Moreover, this model allows us to obtain adequate number of high or low functional avidity CD8^+^ T cells by simply staining with anti-CD45.1 fluorescent antibody for immune signature evaluation.

### Enhanced antigen-specific *in vivo* killing activity in VACV immunized mice

**Figure 3:**
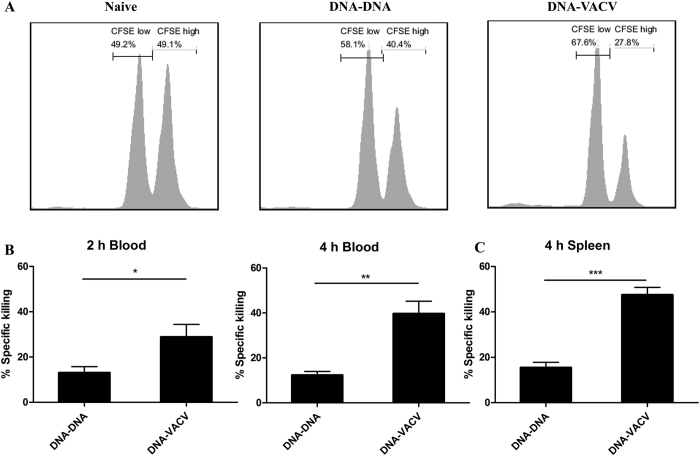
VACV boost increased the cytolytic activity of antigen-specific CD8^+^ T cells. Four weeks after immunization, splenocytes from naïve mice were coated with OVA peptides or left untreated. Peptide-coated cells and non-coated cells were respectively stained with high or low concentration of CFSE, mixed and adoptively transferred into the immunized mice. (**A**) Representative histograms of CFSE-transferred cells in the spleen 4 hr after injection, percentage specific killing of OVA peptide pulsed cells in the blood (**B**) and spleen (**C**).

We further tested whether the enhancement in functional avidity could promote the cytotoxic function of CD8^+^ T cells by *in vivo* killing assay. After adoptive transferring splenocytes pulsed with OVA peptide (CFSE^hi^) or un-treated splenocytes (CFSE^lo^) into immunized mice, we determined *in vivo* killing activity of OVA-specific CD8^+^ T cells in both blood and spleen. Representative histograms of CFSE labeled cells in the spleen 4 hr after transfer are shown in Fig. 3A, the percentage of CFSE^hi^ splenocytes which are target cells of antigen-specific cytotoxic lymphocytes, is much lower in DNA-VACV group than those from DNA-DNA group, demonstrating that the VACV significantly enhanced the cytolytic activity of antigen-specific CD8^+^ T cells. The calculated percentage of *in vivo* killing ability 2 hr/4 hr in the blood and 4 hr in the spleen after transferring are plotted in Fig. 3B,C. Taken together, these data suggest that the improvement in functional avidity of CD8^+^ T cells elicited by VACV mediates the stronger ability of killing activity, although by this method the contribution of increased number of antigen-specific cells to the *in vivo* killing ability could not be excluded.

### The transcriptional profile of high avidity CD8^+^ T cell from VACV immunized mice

**Figure 4:**
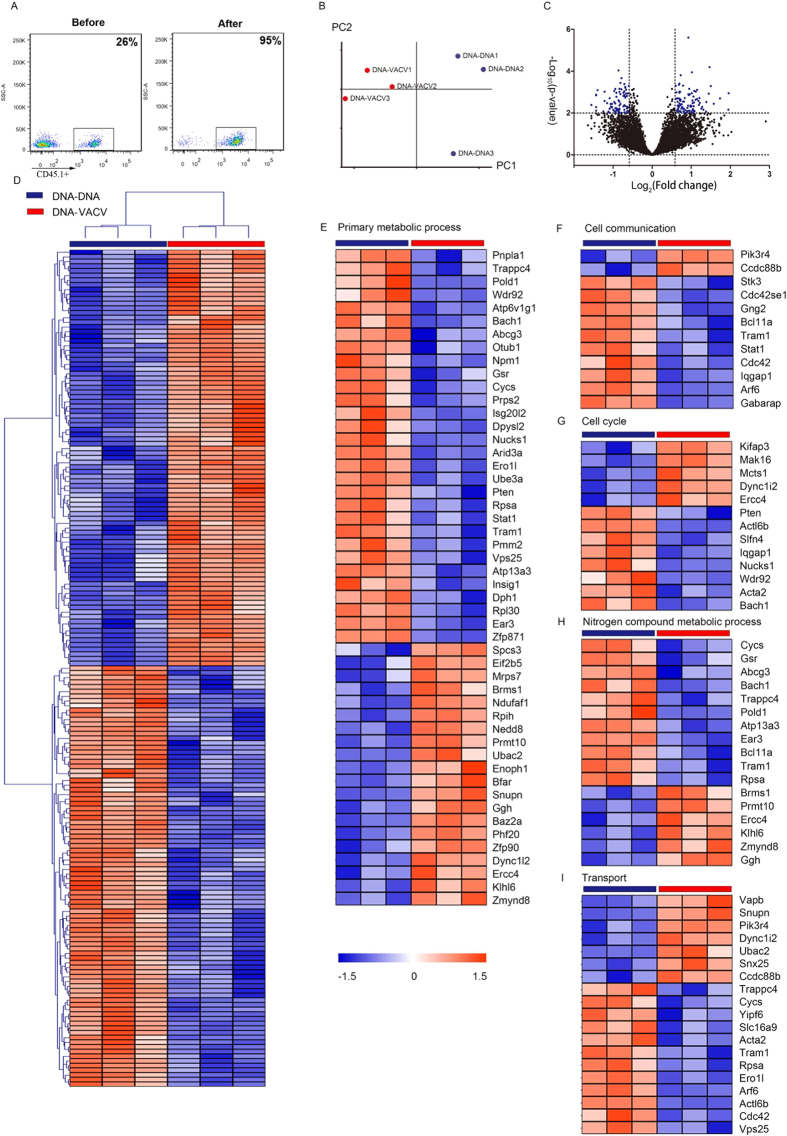
Gene expression profiles of antigen-specific CD8^+^ T cells induced by DNA-DNA and DNA-VACV regimen. (**A**) The purity of CD45.1-PE based cell sorting; (**B**) The PCA plot and (**C**) Volcano plot of gene profiles from DNA-DNA and DNA-VACV groups. (**D**) Supervised hierarchical cluster analysis of differentially expressed genes between cells from DNA-DNA and DNA-VACV groups. Hierarchical cluster analysis of differentially expressed genes involved in primary metabolic process (**E**), cell communication (**F**), cell cycle (**G**), nitrogen compound metabolic process (**H**) and transport (**I**).

**Figure 5:**
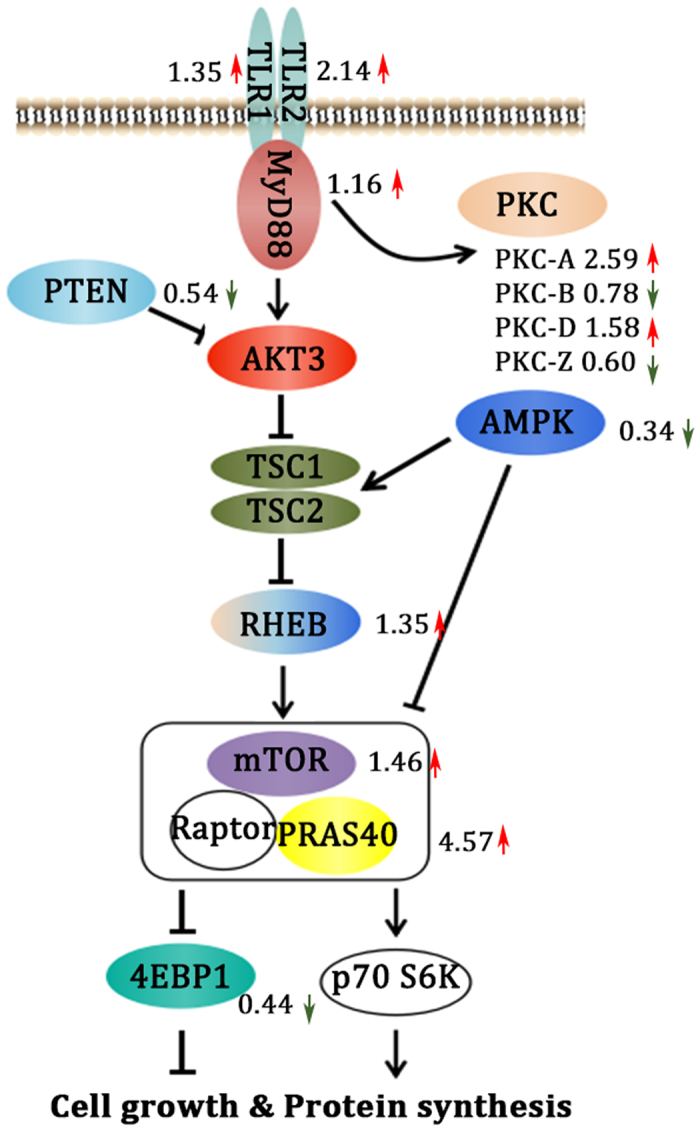
Regulation of MyD88 mediated signaling pathway by VACV boost. The fold change of the key molecules are showed in the right part of corresponding genes. The arrows are in red if up-regulated, whereas down-regulated ones are in green.

To globally characterize the high avidity CD8^+^ T cell from VACV immunized mice and to explore potential molecular mechanism mediates the functional avidity maturation *in vivo*, we analyzed the gene-expression profiles of adoptively transferred CD45.1^+^ OT-I cells induced by different vaccination regimens (GEO Submission: GSE51849). Sorted by CD8^+^ T cells and anti-CD45.1-PE microbeads systems (Fig. 4A), the purified CD45.1^+^ OT-I CD8^+^ T cells from each regimen exhibited a unique signature in the bioinformatics analysis (Fig. 4B–D). The PCA plot revealed a good separation in the principal component space between DNA-VACV and DNA-DNA groups (Fig. 4B). As shown in Fig. 4C,D, 153 genes were differentially expressed between these two groups (P < 0.01, fold change >1.5). We observed that these differentially expressed genes mainly involved in primary metabolic process, transport, nitrogen compound metabolic process, cell cycle and cell communication pathways (Fig. 4E–I and Table 1), indicating these genes might be responsible for the increased levels of functional avidity induced by VACV vaccination. Interestingly, the expression level of MyD88 in antigen-specific CD8 T cells was slightly increased in DNA-VACV group (Fig. 5), and the levels of molecules that positively regulate MyD88-AKT-mTOR signaling pathway was elevated, whereas those negative regulators tend to be suppressed (Fig. 5). Taken together, the present observations suggest that the MyD88 mediated signaling pathway is likely to contribute to the enhanced functional avidity induced by VACV boost, modulating the related molecules might enhance T cell functions and help establishing the dominance of high functional avidity T cells.

### Cytokine profile of CD4^+^ T helper cells, composition of memory subpopulations and cytokine poly-functionality of antigen-specific CD8^+^ T cells are not related to increased T cell functional avidity

**Figure 6:**
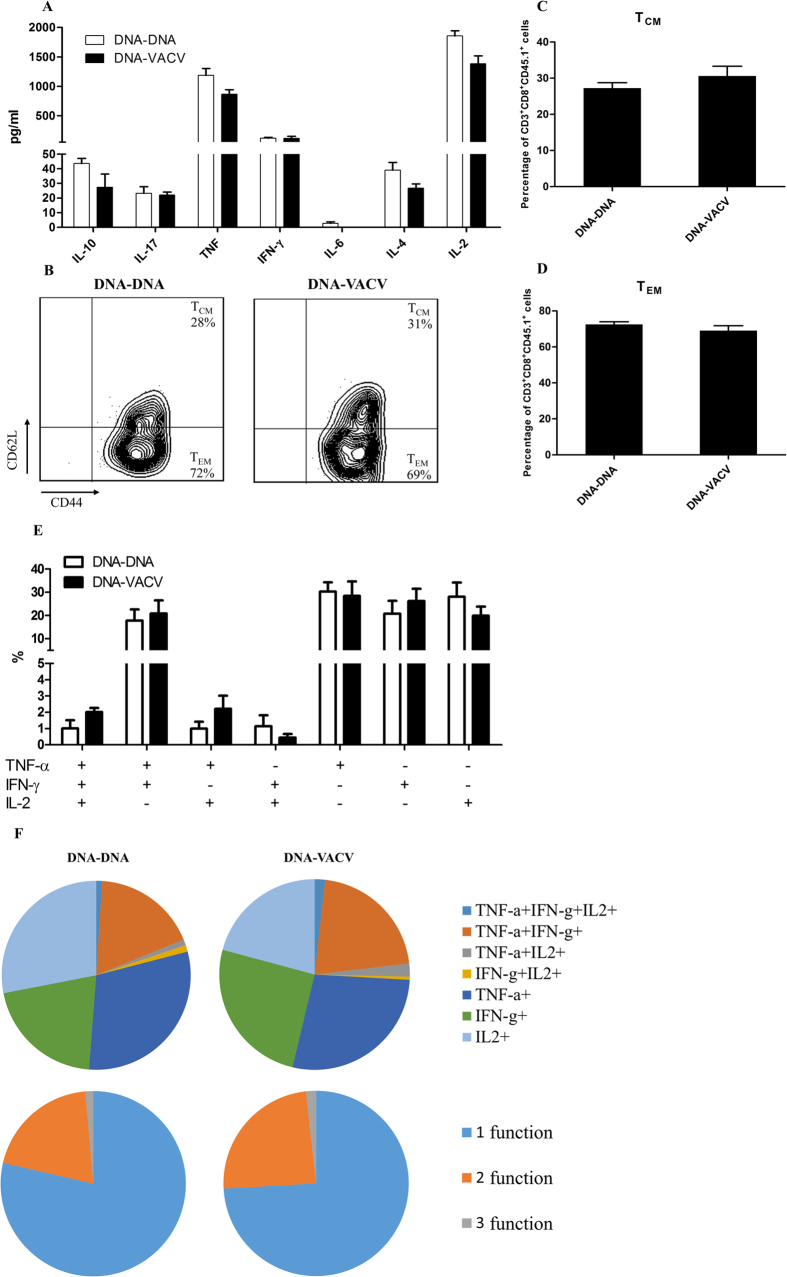
The increased CD8^+^ T cell functional avidity induced by VACV boost was not associated with cytokine profile of CD4^+^ T cells, memory phenotype and poly-functionality of antigen-specific CD8^+^ T cells. To explore the possible association of the functional avidity with other important immune factors, the cytokines profile of CD4^+^ T cells (**A**), the proportion of central and effector memory phenotypes (**B** to **D**), and poly-functionality of antigen-specific CD8^+^ T cells (**E**,**F**) from different vaccination regimens was assessed.

Although we showed that the intrinsic MyD88 mediated pathway of CD8^+^ T cells mainly contributed to the enhanced functional avidity maturation *in vivo*^27^, recent studies suggested that the functional avidity of T cells might be influenced by several factors, such as Th1/Th2/Th17 balance^28^, memory phenotype^29^ and poly-functionality of CD8^+^ T cells^4^,^30^. We therefore tested whether the enhanced avidity is associated with these factors in our model. However, our data showed that the cytokine’s production by the CD4^+^ T cells was identical between the two vaccination regimen groups (Fig. 6A). Similarly, the composition of central memory and effector memory T cells in the antigen-specific CD8^+^ T cells was unaffected by VACV vaccination (Fig. 6B,C,D). In addition, the differences in CD8^+^ T cell poly-functionality did not reach significance (Fig. 6E). Taken together, this data indicate that the improved avidity of T cells induced by VACV is not associated with skewed Th1/Th2/Th17 balance of CD4^+^ helper T cells, the memory phenotype and the poly-functionality of antigen-specific CD8^+^ T cells.

## Discussion

The functional avidity is one of the crucial determinants of T cell functionality. Although the process of primary T cells regulates their sensitivity to peptide antigen *in vitro* is disclosed^19^,^20^,^21^,^22^, how VACV tunes the low avidity CD8^+^ T cells induced by DNA vaccination into high avidity ones *in vivo* is still not defined. Previously, we had proved that the improved functional avidity of T cells induced by VACV is mediated by intrinsic MyD88 mediated signaling pathways in CD8^+^ T cells, instead of antigen presentation efficacy, TCR affinity, or the VACV infection induced MyD88-mediated inflammatory milieu^27^.

In this study, we firstly confirmed the ability of VACV in inducing high functional avidity using different antigens and distinct genetic background mice model. Then, a adoptively transferred model was established by intravenously injecting the monoclonal CD8^+^ T cells from TCR transgenic OT-I mice into wild type C57BL/6 mice to exclude the effect of TCR selection. By evaluating the efficacy of *in vivo* killing activity of antigen-specific CD8^+^ T cells induced by different regimen, we showed that the CD8^+^ T cells with enhanced avidity exerted stronger cytolytic activity. More importantly, this adoptively transferred mice model provides us adequate cells to compare the phenotypes and gene expression profiles between low and high functional avidity antigen-specific CD8^+^ T cells induced by different vaccination regimens.

Recent studies suggest that the T cell functional avidity might be associated with several factors. For example, the argue on whether functional avidity determines poly-functionality of CD8^+^ T cells^4^,^30^. Besides, it has been reported that high avidity T cells employed in anti-tumor therapy tend to secrete Th1 pro-inflammatory cytokines whereas low avidity ones show a Th2 pattern^28^, suggesting that the Th1/Th2/Th17 cell balance might influence the avidity of CD8^+^ T cells. Moreover, it’s believed that the high levels of effector memory CD8^+^ T cells elicited by poxvirus based vaccines are one of the determinants of viral control^29^, indicating that there might be an association between memory phenotype and functional avidity of antigen-specific CD8^+^ T cells induced by poxvirus based vaccines. In this study, however, we demonstrated that increased avidity of CD8^+^ T cells was not correlated with any of these indicators in our system, support our previous hypothesis that the increased avidity induced by VACV is gained and hardwired as intrinsic property of antigen-specific CD8^+^ T cells^27^.

Herein, we show that VACV selectively regulated the expression of several genes in memory antigen-specific CD8^+^ T cells. Specially, transcriptomic analysis suggests that these genes most enriched in the pathways primary metabolic process, transport, nitrogen compound metabolic process, cell cycle and cell communication, which might be contributed to the *in vivo* tuning of functional avidity maturation induced by VACV.

Our previous report showed that MyD88 in T cells is essential in VACV boosted T cell functional avidity^27^, herein, the microarray data showed that the expression of MyD88 in antigen-specific CD8 T cells was slightly increased (fold change = 1.16, Fig. 5) in DNA-VACV group. We also noticed that Pik3r4, (Phosphoinositide 3-kinase regulatory subunit 4, also known as Pi3kr4), a regulatory subunit of the PI3K complex that mediates formation of phosphatidylinositol 3-phosphate, was significantly up-regulated in DNA-VACV group (Fig. 4F). The PI3K-Akt-mTOR signaling pathway is essential in MyD88 mediated CD8 T cell clonal expansion and memory formation following VACV infection^31^,^32^,^33^. Consistent with these findings, the expression levels of positive regulators such as *TLR1/2*, *RHEB*, *mTOR* and *PRAS40* in this signaling network were increased, whereas the negative regulators like *AMPK* and *4EBP1* were reduced in VACV boost group (Fig. 5). In addition, *PTEN*, a negatively regulator of PI3K-Akt signaling pathway^34^, was down-regulated in the DNA-VACV group (Figs 4F and 5). These evidence together suggest that VACV mediated functional avidity maturation might go through MyD88-PI3K-Akt-mTOR-PTEN pathway and modulating the corresponding genes would enhance T cell functions and help establishing the dominance of high functional avidity T cells.

In line with our findings that VACV increased the frequency of antigen-specific CD8 T cells with enhanced avidity, overexpression of *PTEN* results in apoptosis^35^, whereas suppression of *PTEN* expression prevents apoptosis^36^. Thus, VACV boost might also prolong the survival of antigen-specific CD8 T cells though modulating the expression of *PTEN*. Converging *BFAR* (Bifunctional apoptosis regulator), which bears anti-apoptotic activity, was selectively up-regulated, whereas *WDR92*, an apoptosis-inducing gene, was down-regulated in DNA-VACV group also supports CD8 T cells boosted by VACV are resistant to apoptosis. Moreover, VACV boost could also mediated metabolic process by selectively increasing the expression of positive regulators such as *EIF2B5*, *NDUFAF1*, *NEDD8*, *SNUPN*, *GGH*, *BAZ2A*, *PHF20*, *DYNC1I2* and *ZMYND8*, and decreasing the expression of molecules involved in protein degradation like *UBE3A* and *INSIG1*, the cells might be in a state ready to activation upon encountering re-stimulators. Taken together, these data suggest that VACV boost increased the numbers of antigen-specific CD8 T cells with an increased metabolic process and extended lifespan.

In summary, we provide an *in vivo* model of inducing high functional avidity T cells and the method of analyzing those antigen-specific CD8^+^ T cells. Moreover, our data demonstrate that the VACV has the ability to tune the low level of functional avidity of antigen-specific CD8^+^ T cells primed by DNA vaccination into higher ones, which is associated with the enhanced CTL *in vivo* killing activity, but is not correlated with cytokine profile of CD4^+^ T helper cells, memory phenotype and poly-functionality of antigen-specific CD8^+^ T cells. Global gene expression analysis suggests that VACV selectively activated a unique set of genes that mainly involved in several signaling pathways, which might be responsible for the functional avidity maturation *in vivo*. Taken together, these results offer insights into the regulatory mechanisms of *in vivo* tuning function avidity.

## Materials and Methods

### Recombinant DNA vaccines and VACV vaccines

The vector pSV1.0 contains the cytomegalovirus immediate-early promoter and a 72-bp element of simian virus 40 enhancer was used as the DNA vaccine vector. The pSV1.0-*gagenv* (DNA-*gagenv*), encoding *gag* and *envelop* fusion gene of HIV-1 CRF01_AE2f. The pSV1.0-*ova* (DNA-*ova*), encoding the full length of chicken ovalbumin gene. These DNA vaccines were extracted with Qiagen EndoFree Plasmid Maxi Kit and eluted in pyrogen-free deionized water. VACV used in this study is derived from Chinese Tiantan strain. VACV-*gagenv* expresses HIV-1 AE Gag and Env proteins, and VACV-*ova* expresses OVA protein were propagated in Vero cell line.

### Mice and immunization

All animals’ experimental protocol were reviewed and approved by the Institutional Animal Care and Use Committee of Shanghai Public Health Clinical Center and were performed in accordance with relevant guidelines and regulations. Six-week-old female C57BL/6 mice were primed with 1 doses of 100 μg DNA-*gagenv* and boosted with either 100 μg DNA or 10^7^ plaque-forming units VACV-*gagenv* at 2 weeks post-prime (Fig. 1A). In adoptive transfer experiments, 6-week-old female C57BL/6 mice (CD45.2^+^) received 10^6^ OT- I CD8^+^ T cells (CD45.1^+^) and were inoculated with vaccines expressing OVA as shown in Fig. 3A. All mice were immunized in the quadriceps muscle, with total 100 μl in volume. OT- I mice used in this study is derived from C57BL/6 background.

### Splenocytes preparation and CD8^+^ T (CD4^+^ T) cells isolation

Splenocytes were isolated by mechanical disruption of spleens and filtrated through mesh gauze, the cells were then treated with red blood cells lysis buffer. CD8^+^ T cells or CD4^+^ T cells were subjected to negative selection using Miltenyi Mouse CD8a^+^ T Cell Isolation Kit or Mouse CD4^+^ T Cell Isolation Kit, respectively, according to the manufacturer’s instruction. Purity of sorted cell populations was at least 95%, determined by flow cytometer (FACS Aria II, BD Biosciences).

### ELISpot assay and IFN-γ secreting functional avidity assay

ELISpot assays were performed according to IFN-γ ELISpot Kit instruction (BD Biosciences). Briefly, 96-well plates were coated with anti-mouse IFN-γ antibodies at 4 °C overnight, then washed and blocked with R10 medium (RPMI-1640 containing 10% FBS and 1% Penicillin & Streptomycin) at room temperature for 2 hr. A serial 10-fold dilution of peptide and isolated splenocytes were added at 2 × 10^5^ cells per well. The plates were incubated at 37 °C and 5% CO_2_ for 20 hr. After incubation, cells were removed and plates were washed with PBST, then treated with anti-mouse IFN-γ biotinylated detection antibodies, and incubated at 37 °C for 2 hr, then washed again with PBST and treated with streptavidin-horserradish superoxidase conjugated anti-biotin antibodies and incubated for 1 hr at room temperature. Unbounded antibodies were removed by rinsing the plates with PBST and PBS. Then, AEC substrate solution (BD Biosciences) was added and incubated for 30 min before rinsing away with water. Plates were air-dried and the spots were counted with Immunospot Reader (Champspot III, Beijing Sage Creation Science, China). Spot forming cells (SFCs) at each stimulating concentration of peptide were normalized by maximal value (The max SFCs induced by the saturating concentration of peptides), and variable slope sigmoid regression was used to infer the effective concentration 50 (EC_50_), which is the peptide concentration required to generate 50% maximal cytokine production. All the functional avidity assay in this study is based on IFN-γ production. Peptides were synthesized by GL Biochem (Shanghai, China) with >95% purity. Peptides used in ELISpot assay were Env203 (AISLLNATAIAVAGW) and Gag37 (QPLSPRTLNAWVKVV), which were reported as a dominant and a sub-dominant epitopes respectively in our previous report^37^ and confirmed by intracellular cytokine staining (ICS, data not shown), the dominant epitope OVA (SIINFEKL) derived from OVA protein were used for OVA based vaccines evaluation^38^.

### Th1/Th2/Th17 cytokines expression pattern using a cytometric bead array

Cytokine profiles were determined using the Mouse Th1/Th2/Th17 Cytokine Kit following the manufacturer’s instructions (BD Biosciences). Briefly, 2 × 10^5^ purified CD4^+^ T cells were stimulated in presence of PMA (50 ng/ml) and ionomycin (1 μg/ml) at a final volume of 100 μl. After incubation for 20 hr, the cells were centrifuged and 50 μl supernatant was mixed with 50 μl capturing beads and 50 μl PE detection reagent of the kit, then incubated for 2 hr at room temperature in the dark. Samples were washed and re-suspended in 200 μl wash buffer and analyzed by flow cytometry (BD Bioscience). Standard curves were generated for each cytokine using a mixed bead standard to quantify cytokine concentrations.

### Antibodies and intracellular staining

A total of 10^6^ splenocytes were stimulated with the peptides (5 μg/ml) at 37 °C for 6 hr. One hr after addition of peptides, brefeldin A (eBioscience) and monensin (eBioscience) were added at 1 μg/ml and 1 μM, respectively. The stimulated cells were stained with a cocktail of surface antibodies on ice for 30 min and then subjected to fixation and permeation with fix/perm buffer (BD Bioscience). Antibodies against intracellular cytokines were added, and cells were incubated on ice for another 30 min. After washing with PBS containing 2% newborn calf serum, the cells were fixed in 2% paraformaldehyde and then acquired by flow cytometry (FACS Aria II, BD Bioscience).

The following antibodies were used in this study: CD3-PerCP (clone 145-2C11), CD8-PB (clone 53-6.7), CD44-FITC (clone 1M7), CD62L-PE-Cy7 (clone MEL-14), TNF-α-PE-Cy7 (clone MP6-XT22), IL-2-APC (clone JES6-5H4), IFN-γ-PE (clone XMG1.2), all from BD Biosciences.

### *In vivo* cytolytic assay

The *in vivo* cytolytic assay was measured as described elsewhere with modifications^39^. Briefly, C57BL/6 splenocytes were coated with 1 μM OVA peptide (SIINFEKL) or left untreated at 37 °C for 1 hr. After washing, peptide coated target cells were labeled with 5 μM CFSE (CFSE^hi^), whereas uncoated control cells were labeled with 0.8 μM CFSE (CFSE^lo^ cells). Then, a mixture of 4 × 10^6^ CFSE^hi^ cells and 4 × 10^6^ CFSE^lo^ cells were adoptive transferred into naïve or immunized mice. Killing of peptide coated target cells were detected in the blood 2 hr and 4 hr and in the spleen at 4 hr after adoptively transfer, by analyzing CFSE-labeled cells. The percentage of specific cell lysis was then calculated by using the formula:


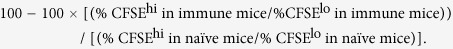


### Transcriptional profile of antigen-specific CD8^+^ T cells

CD45.1-PE antibody (clone A20, Biolegend) and anti-PE MicroBeads (Miltenyi) were used for enrichment of congenically marked OVA-specific CD45.1^+^ OT-I CD8^+^ T cells. Total RNA was extracted by the RNeasy Mini Kit (Qiagen). Followed by amplification and biotin labeling, the samples were hybridized using Illumina Total Prep RNA Amplification Kit (Ambion). Mouse WG-6v2 Expression BeadChips were used for analysis of transcriptome. Data were analyzed using the BRB-Array Tools version 4.3.2 stable (http://linus.nci.nih.gov./BRB-ArrayTools.html). Log2-transformed data were normalized by the results of median normalization. Exclude the spot if the intensity is below 16 and exclude a gene if less than 20% of expression data values have at least a 1.2-fold change. Using selection criteria of P < 0.01 and fold change of 1.5 or greater, differentially expressed genes between groups were determined by univariate *t* test. Principal Component Analysis (PCA) was performed with all the genes after normalization and fitering using the online tool “NIA Array Analysis tool” (http://lgsun.grc.nia.nih.gov/ANOVA/index.html). Hierarchical cluster analysis were performed using Multiexperiment Viewer (MeV 4.9). Gene ontology was implemented by Gene Ontology Consortium (http://geneontology.org/), a web-based computational tool.

### Statistical analysis

The cytometric bead array data was generated in a graphical and tabular format using the BD cytometric bead array analysis software. The difference between groups was performed by unpaired *t* test using the GraphPad software.

## Additional Information

**How to cite this article**: Hu, Z. *et al*. Immune Signature of Enhanced Functional Avidity CD8^+^ T Cells *in vivo* Induced by Vaccinia Vectored Vaccine. *Sci. Rep.* **7**, 41558; doi: 10.1038/srep41558 (2017).

**Publisher's note:** Springer Nature remains neutral with regard to jurisdictional claims in published maps and institutional affiliations.

## Figures and Tables

**Table 1:** The GO items of differentially expressed genes.

Biological process	No. of genes
Primary metabolic process (GO:0044238)	50
Transport (GO:0006810)	18
Citrogen compound metabolic process (GO:0006807)	17
Cell cycle (GO:0007049)	13
Cell communication (GO:0007154)	12
Biosynthetic process (GO:0009058)	13
Cellular component organization (GO:0016043)	12
Phosphate-containing compound metabolic process (GO:0006796)	11
Catabolic process (GO:0009056)	8
Regulation of biological process (GO:0050789)	7
